# Priming Soybean cv. Primus Leads to Successful Systemic Defense Against the Root-Lesion Nematode, *Pratylenchus penetrans*

**DOI:** 10.3389/fpls.2021.651943

**Published:** 2021-05-12

**Authors:** Shimaa Adss, Benye Liu, Ludger Beerhues, Volker Hahn, Holger Heuer, Ahmed Elhady

**Affiliations:** ^1^Institute for Epidemiology and Pathogen Diagnostics, Julius Kühn-Institute, Federal Research Centre for Cultivated Plants, Braunschweig, Germany; ^2^Institute of Pharmaceutical Biology, Technische Universität Braunschweig, Braunschweig, Germany; ^3^State Plant Breeding Institute, Universität Hohenheim, Stuttgart, Germany; ^4^Department of Plant Protection, Faculty of Agriculture, Benha University, Benha, Egypt

**Keywords:** induced systemic resistance, defense priming, plant-parasitic nematode, *Pratylenchus*, *N*-acyl-homoserine lactone, phytoalexin, *Ensifer meliloti*, *Glycine max*

## Abstract

Root lesion nematodes, *Pratylenchus penetrans*, are major pests of legumes with little options for their control. We aimed to prime soybean cv. Primus seedlings to improve basic defense against these nematodes by root application of *N*-3-oxo-tetradecanoyl-*L*-homoserine lactone (oxo-C14-HSL). The invasion of soybean roots by *P. penetrans* was significantly reduced in plants that were pre-treated with the oxo-C14-HSL producing rhizobacterium *Ensifer meliloti* strain ExpR+, compared to non-inoculated plants or plants inoculated with the nearly isogenic strain *E. meliloti* AttM with plasmid-mediated oxo-C14-HSL degradation. The nematodes were more clustered in the root tissues of plants treated with the AttM strain or the control compared to roots treated with the ExpR+ strain. In split-root systems primed on one side with strain ExpR+, root invasion was reduced on the opposite side compared to non-primed plants indicating a systemic plant response to oxo-C14-HSL. No additional local effect was detected, when inoculating nematodes on the ExpR+ primed side. Removal of oxo-C14-HSL after root exposure resulted in reduced root invasion compared to non-primed plants when the nematodes were added 3, 7, or 15 days later. Thus, probably the plant memorized the priming stimulus. Similarly, the plants were primed by compounds released from the surface of the nematodes. HPLC analysis of the root extracts of oxo-C14-HSL treated and untreated plants revealed that priming resulted in enhanced phytoalexin synthesis upon *P. penetrans* challenge. Without root invading nematodes, the phytoalexin concentrations of primed and non-primed plants did not significantly differ, indicating that priming did not lead to a persistently increased stress level of the plants. Upon nematode invasion, the phytoalexins coumestrol, genistein, and glyceollin increased in concentration in the roots compared to control plants without nematodes. Glyceollin synthesis was significantly more triggered by nematodes in primed plants compared to non-primed plants. The results indicated that the priming of soybean plants led to a more rapid and strong defense induction upon root invasion of nematodes.

## Introduction

In their natural environment, plants are continuously exposed to a wide range of biotic stresses and evolved defense strategies that presumably harness associated microbes (Liu et al., 2020). In agriculture, pathogens and pests severely lower crop productivity and influence food security. Plant-parasitic nematodes are among the major limiting factors of crop production worldwide (Jones et al., 2013). The root lesion nematodes, *Pratylenchus* spp., invade roots, cause lesions by migrating through root tissues, only feed on living root cells, and lay eggs within the root or in soil. The root lesion nematodes are a major problem for legumes due to high reproduction rates on these hosts. They are abundant in soybean fields (Elhady et al., 2018, 2019, 2020).

Priming crop plants for enhanced defense against pathogens could become an environmentally friendly strategy for plant protection (Borges et al., 2014; Conrath et al., 2015; Wehner et al., 2019). Priming of plants by an inducer results in a faster and stronger response of the plant defense upon pathogen attack. Such a fast defense response is crucial for the plant to resist nematode attack because effectors of these pathogens suppress plant defenses during establishment in the root (Vieira et al., 2018). The priming state is preserved for a period after removal of the priming stimulus, and sometimes it is even transgenerationally transmitted (Ramírez-Carrasco et al., 2017). Ideally, it is not associated with a cost for the plant after the initial phase of priming because the stimulus does not trigger an ongoing defense reaction (Martínez-Medina et al., 2016).

Defense priming can be induced by different chemical compounds such as β-aminobutyric acid, salicylic acid, pipecolic acid, jasmonic acid, or volatile organic compounds (Navarova et al., 2012; Zhong et al., 2014; Ji et al., 2015; Hu et al., 2017; Thevenet et al., 2017). Plants may also be primed by beneficial soil organisms such as rhizobacteria or fungi (Jung et al., 2012; Molinari and Leonetti, 2019). Pathogen-associated molecules have been used to induce defense priming, among them are peptides, polysaccharides, and lipids that are perceived by plants through pattern recognition receptors (Dodds and Rathjen, 2010). For nematodes, ascarosides were reported to be perceived by plants and were shown to trigger plant defense responses (Klessig et al., 2019), but were not tested for priming of plant defense.

Recently, several reports suggested that bacterial quorum sensing signaling molecules like the *N*-acyl-homoserine lactones induce plant defense responses like callose deposition in the cell wall, accumulation of oxylipins, and stomata closure (Zarkani et al., 2013; Schenk and Schikora, 2014; Shrestha et al., 2019). The induction of defense against powdery mildew in barley by *N*-3-oxo-tetradecanoyl-*L*-homoserine lactone (oxo-C14-HSL), produced by *Ensifer meliloti*, was genotype-dependent (Shrestha et al., 2019). However, it was not investigated whether enhanced defense of the plant persisted over longer periods after removal of the inducer oxo-C14-HSL or the bacterial strain that produced it, or whether it enhances defense of roots against soilborne diseases. Investigations to prime plants for defense against plant-parasitic nematodes are rare. Rice plants treated with β-aminobutyric acid (Ji et al., 2015) or silicon (Zhan et al., 2018) reduced the invasion of *Meloidogyne graminicola* by activation of basal defenses as indicated by callose deposition and accumulation of reactive oxygen species (ROS), but defense priming of rice was not investigated.

In this study, we tested whether soybean cv. Primus plants are primed by root exposure to oxo-C14-HSL to better defend against a later attack of the plant-parasitic nematode *Pratylenchus penetrans*. We investigated whether and for how long an enhanced defense persisted after removal of the stimulus. The effect by the rhizobacterial inducer oxo-C14-HSL was compared to that of surface-released compounds of the nematode as priming agent. We hypothesized that the priming effect of oxo-C14-HSL on plant defense against nematode invasion is mediated by a systemic plant response rather than by a local response of the tissue exposed to oxo-C14-HSL, or by a direct effect of oxo-C14-HSL on the nematodes. This was investigated in split-root systems. Furthermore, we analyzed the accumulation of phytoalexins as a measure of plant defense induction after root exposure to oxo-C14-HSL, and after invasion of nematodes into roots of either primed or non-primed plants. The study provided a proof of concept for primability of soybean plants for defense against plant-parasitic nematodes to pave the way for further investigations on how to enhance the effect with respect to responsiveness of plant genotypes and the efficiency of different priming agents or microbes.

## Materials and Methods

### Plants, Bacteria, and Nematodes

Soybean (*Glycine max*) cv. Primus seeds were thoroughly washed with water. Each washed seed was surface disinfected with 1.5% sodium hypochlorite for 5 min and then washed with sterile water three times for 1 min and once for 10 min. The seeds were placed on moist sterilized paper towels in Petri dishes for germination. After 5 days, equally developed seedlings were selected and three of them were grown in a jar containing 60 ml of 1/2 MS medium. Plants were allowed to grow for additional 3 days in controlled conditions: day/night 16/8 h and 18/16∘C, the light intensity of 150 μmol/m^2^ s, and 60% humidity, in a growing chamber.

Mixed stages of *P. penetrans* were extracted from 2-month-old axenic cultures on carrot discs through a Baermann funnel (European and Mediterranean Plant Protection Organization, 2013). Nematodes were surface disinfected on 5-μm sieves (CellTrics, Sysmex, Norderstedt, Germany) by soaking in 0.02% HgCl_2_ for 3 min, 4,000 ppm streptomycin sulfate for 3 min, and CellCultureGuard (PanReac AppliChem, Darmstadt, Germany) for 4 h. Finally, the nematodes were washed on the sieve and incubated overnight in sterilized tap water. The concentration of nematode suspension was calibrated using a counting chamber to have a final suspension containing 500 nematodes per ml. Inoculation of plants with 1,000 *P. penetrans* was done by digging four 5 cm deep half an-inch-wide holes in 2 cm distance around the shoot, and equally distributing the nematode suspension.

*Ensifer meliloti* Rm2011 ExpR+ (strain ExpR+) and *E*. *meliloti* Rm2011 pBBR2-attM (strain AttM) carrying the lactonase gene *attM* from *Agrobacterium tumefaciens* on the plasmid vector pBBR1MCS-2 (Pellock et al., 2002; Zarkani et al., 2013) were grown in TY medium (Beringer, 1974) until the OD_600 nm_ reached 0.6–0.8. Bacterial cultures were centrifuged at 4,000 × *g* for 10 min and resuspended in 10 mM MgCl_2_. The roots of soybean plants were inoculated three times over 2 weeks with 10 ml suspensions of strain ExpR+ or strain AttM in 10 mM MgCl_2_ (OD_600 nm_ 0.1), or 10 mM MgCl_2_ as control.

### Priming of Soybean With oxo-C14-HSL for Defense Against *P. penetrans*

To explore and confirm the effect of oxo-C14-HSL (Sigma-Aldrich, Munich, Germany) on the priming of soybean cv. Primus defense toward *P. penetrans*, both the oxo-C14-HSL producing rhizobacterium *E. meliloti* strain ExpR + and the pure compound oxo-C14-HSL were used. Five-day-old soybean seedlings were planted in 8 cm × 6.2 cm pots filled with 200 ml autoclaved sand as an artificial growth substrate. The roots of the soybean plants were drenched three times over 2 weeks with a 10 ml suspension of *E. meliloti* strain ExpR+ in 10 mM MgCl_2_. The same density and volume of strain AttM was used as bacterial control, and 10 mM MgCl_2_ as solvent control. This resulted in three treatments including control, *E. meliloti* AttM, and *E. meliloti* ExpR+. Each treatment represented 20 biological replicates from two independent experiments.

As for synthetic oxo-C14-HSL, soybean seedlings of cv. Primus were first induced *in vitro*. A 60 mM stock solution of oxo-C14-HSL was prepared in acetone and diluted with sterile deionized water to a 6 μM working solution. For priming of soybean plants, roots were treated *in vitro* for 5 days with 6 μM oxo-C14-HSL. Plants were transplanted to pots in the greenhouse and left for 3 days to adapt to the conditions. Mixed stages of *P. penetrans* were prepared as described to inoculate 1,000 to each pot. One week later, plants were sampled. Shoot and root fresh weights were measured. Nematodes were stained in cleared roots with 1% acid fuchsin and microscopically counted (Bybd et al., 1983). In order to analyze the spatial dispersion of nematodes within primed and non-primed plants, roots were stained, sectioned into 1.2 cm pieces, and 25 pieces from each plant were randomly selected to determine the index of dispersion *I*_D_ = (*n*–1) ^∗^ variance/mean (Iwao, 1968), with *n* = number of analyzed root sections per plant. *I*_D_ was used to calculate the coefficient *Z*=sqrt (2*I_D_) -*sqrt*(2* (*n*−1)−1) (Ghaderi et al., 2018) and to statistically compare among the three treatments including control, *E. meliloti* AttM, and *E. meliloti* ExpR+. Each treatment represented 10 biological replicates. The nematodes were quantified and visualized using a stereomicroscope (Olympus Microscope SZX12) and photographed with a Jenoptik ProgRes^®^ digital camera. Images were recorded using CapturePro 2.8 software (Jenoptic, Jena, Germany).

### Priming by oxo-C14-HSL Compared to DL-β-Aminobutyric Acid (BABA) and Surface-Released Compounds of *P. penetrans* (PpNemawater)

We investigated to what extent oxo-C14-HSL can prime the defense of soybean cv. Primus compared to the known priming agent BABA, or PpNemawater. The surface released compounds were derived from *P. penetrans* according to Mendy et al. (2017) with slight modifications: Approximately 300,000 freshly hatched juveniles of *P. penetrans* were incubated in 10 ml sterile water for 48 h at room temperature with continuous rotation. After centrifugation at 800 × *g* for 3 min, the supernatant was filtered through a 20-μm filter and referred as PpNemawater. Plants were treated *in vitro* for 5 days with 6 μM oxo-C14-HSL, 25 mM BABA, or 5 ml PpNemawater. Sterile water was used as a control. Plants were transplanted to a pot system in the greenhouse and infected with 1,000 *P. penetrans* after 3 days. Nematodes were counted 10 days post-infection. This resulted in four treatments with 10 replicates per treatment.

### Direct and Plant Mediated Effect of oxo-C14-HSL on *P. penetrans*

To investigate whether the effect of oxo-C14-HSL on nematode invasion is a local effect or mediated by a systemic response, a split-root experiment (Dababat and Sikora, 2007) was done in the greenhouse. Three square pots of 7 cm × 7 cm × 8 cm were arranged as follows: Two pots were attached to each other and one pot was placed in the center above those two pots. Two-week-old seedlings of soybean were transplanted in the center of the upper pot, which was half filled with sterile sand. Roots were divided into two halves. One-half of each split-root was inoculated three times with *E. meliloti* ExpR+. Infective stages of *P. penetrans* were inoculated either to the side of the roots colonized by strain ExpR+ or to the opposite side, with 700 nematodes of mixed stages per pot. In controls, only nematodes and no bacteria were inoculated to one of the split-roots. After 7 days, the shoot of each plant and the roots from both sides were harvested and weighed. Roots from the side of nematode inoculation were stained with acid fuchsin to microscopically count invaded nematodes (Bybd et al., 1983). This resulted in three treatments including control, local, and systemic response. Each treatment was represented by 10 biological replicates.

To further explore any direct effect of oxo-C14-HSL on *P. penetrans*, we used *in vitro* assays to analyze the mortality of the nematodes induced by the bacterial strain ExpR+, strain AttM, or oxo-C14-HSL. Cultures of the bacterial strains as mentioned previously in Section “Plants, Bacteria, and Nematodes” and a 6 μM solution of oxo-C14-HSL were prepared. As controls, 10 mM MgCl_2_ (to explore the effect of bacterial strains), and a solution of sterile water containing 25 μl acetone (to explore the effect of molecules) were used. In a 96-well plate, 200 freshly extracted *P. penetrans* were added to 250 μl of the up-mentioned solutions and incubated at room temperature. Total and dead nematodes were counted after 48 h using a stereomicroscope (Olympus Microscope SZX12) to calculate the mortality as a percentage. This resulted in five treatments including ExpR+, AttM strains, oxo-C14-HSL, 10 mM MgCl_2_, and diluted acetone as controls. Each treatment was represented by 24 replicates.

### Persistence of Plant Defense After Removal of Inducers

Soybean seedlings of cv. Primus growing on 1/2 MS medium under sterile conditions were treated with oxo-C14-HSL for 5 days at a final concentration of 10 μM for priming induction. The oxo-C14-HSL solution was added in a reservoir in the middle of a jar with three plants around it with their roots in the reservoir. In controls, the reservoir was filled with the solvent control. Other samples were treated with 5 ml of PpNemawater. Plants were allowed to grow for an additional 5 days at the same conditions: light/dark 16/8 h, 18/16∘C, light intensity 150 μmol/m^2^ s, 60% humidity. Plant roots were exposed to oxo-C14-HSL or PpNemawater for 5 days. Plants were taken from the jars, roots were gently washed with water to remove the inducer, and plants were transplanted into sterile sand and kept for 3, 7, or 15 days before inoculation of 750 *P. penetrans*. Roots were harvested 7 days after inoculation to quantify invaded nematodes by staining with 1% acid fuchsin and microscopic counting (Bybd et al., 1983). This experiment was done with 9–14 biological replicates.

### Analysis of Phytoalexins by HPLC

Soybean roots were harvested 5 days after the treatment with oxo-C14-HSL and inoculation of nematodes. Roots (0.25 g) were kept at −20∘C and homogenized using a TissueLyser II (Qiagen, Germany) with a 3 mm glass bead at 30 Hz for 5 min. Phytoalexins were extracted with five volumes of 80% methanol at 20∘C for 12 h. After centrifugation at 12,000 *g* for 10 min, the supernatants were filtered through a 0.45-μm membrane before HPLC analysis. A Waters 2795 Seperatons Module coupled with Waters 2996 Photodiode Array Detector was operated with Software Empower Pro 2002. A Water symmetry C8 column (4.6 × 150 mm) was used for separation. The mobile phase was water with 0.1% formic acid (A) and acetonitrile (B). The separation program was as follows: starting with 5% B for 2 min with a flow rate of 0.5 ml/min, then a linear gradient of acetonitrile from 5 to 60% in 30 min. The injection volume was 20 μL. Glyceollin and coumestrol were detected at 285 or 343 nm, respectively.

### Quantification of Total Phenolics

To quantify the total phenolic compounds in roots of primed and non-primed soybean plants in response to nematode attack, roots of soybean plants were collected 3 days after nematode inoculation. Plants without nematode infection were used as control. Roots were frozen with liquid nitrogen, pulverized in a TissueLyser II (Qiagen) with a 3 mm glass bead at 30 Hz for 5 min. The total phenolic compounds in 1 g were quantified by a colorimetric assay using the Folin-Ciocalteu method (Ainsworth and Gillespie, 2007). Gallic acid (Sigma-Aldrich, Darmstadt, Germany) was used as a reference for the quantification by spectrophotometry at 765 nm.

### Statistical Analysis

Analysis of variance was done using the procedure GLIMMIX of the statistical software SAS 9.4 (SAS Institute Inc., Cary, NC, United States) to fit generalized linear models. For count data and the dispersion coefficient *Z*, the procedure was used with the assumption of a Poisson distribution with a log link function, and the Kenward–Roger’s procedure was used to estimate degrees of freedom. For multiple comparisons, the *p*-value was adjusted by the method of Tukey. Graphs were generated using Prism 7 (GraphPad Software, La Jolla, CA, United States).

## Results

### Rhizosphere Inoculation of Soybean Plants With *E. meliloti* ExpR+ Producing oxo-C14-HSL Reduced Root Invasion of *P. penetrans*

**FIGURE 1:**
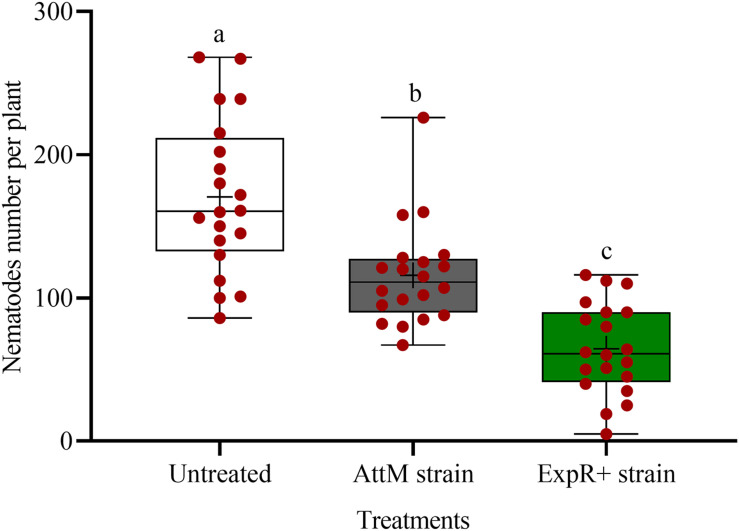
Effect of previous plant priming with the oxo-C14-HSL-producing *Ensifer meliloti* ExpR + strain on the invasion of *Pratylenchus penetrans* into soybean roots. The lactonase-expressing strain *E. meliloti* AttM and MgCl_2_ were used as controls without oxo-C14-HSL priming. Invaded nematodes were stained and counted 10 days post-infection. Different letters indicate significant differences among treatments (GLM, Poisson distribution and log link, Tukey adjustment, *n* = 20).

**FIGURE 2:**
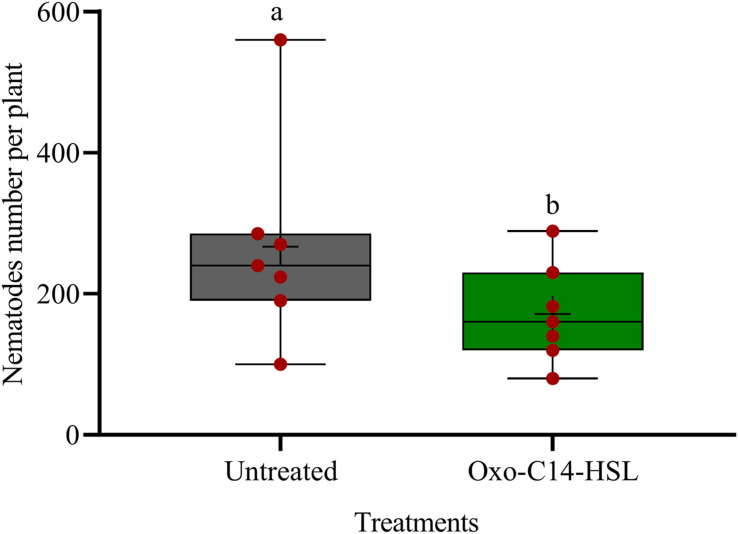
Effect of synthetic oxo-C14-HSL as priming agent on *Pratylenchus penetrans* invasion into soybean roots. Different letters indicate significant differences among treatments (GLM, Poisson distribution and log link, Tukey adjustment, *n* = 7).

We investigated the potential of bacteria producing oxo-C14-HSL in the rhizosphere to enhance the defense of soybean plants toward *P. penetrans*. Significantly fewer nematodes invaded the roots of plants that were exposed to the oxo-C14-HSL-producing strain *E. meliloti* ExpR+ compared to control plants, or compared to plants inoculated with the lactonase-expressing strain *E. meliloti* AttM that does not accumulate oxo-C14-HSL (Figure 1). This suggested that oxo-C14-HSL induced plant defense and thereby reduced nematode invasion into roots. A less pronounced reduction of the invasion of *P. penetrans* was observed in soybean roots that have been treated with the AttM strain compared to the control (Figure 1). This can be attributed to a slight defense induction by bacteria associated molecular patterns to which plants usually respond. Moreover, we tested the commercially available form of oxo-C14-HSL to confirm that the priming effect is due to this signaling molecule. Plants were treated either with oxo-C14-HSL *in vitro* or with the acetone control. The invasion of *P. penetrans* was significantly reduced in the oxo-C14-HSL treated roots compared to the control (Figure 2). Plant growth was not significantly changed by oxo-C14-HSL compared to control plants within the period of the experiment (Supplementary Figure 1).

**FIGURE 3:**
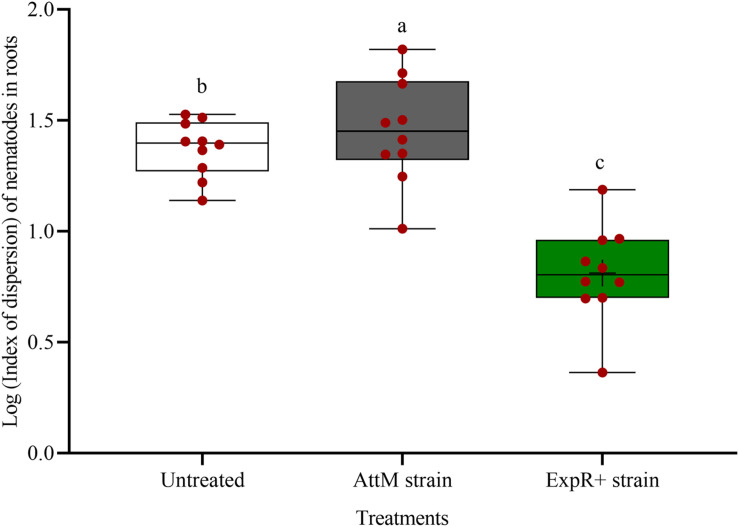
Spatial dispersion of invaded *Pratylenchus penetrans* inside the roots of soybean plants primed with *Ensifer meliloti* ExpR + producing oxo-C14-HSL compared to roots of non-primed soybean inoculated with the lactonase-expressing strain *E. meliloti* AttM, or MgCl_2_ as control. The coefficient of dispersion Z of *P. penetrans* was compared to a random (Poisson) distribution using the GLIMMIX procedure. (Tukey adjustment, log link, *n = 10*). Different letters indicate significant differences among treatments.

The spatial dispersion of *P. penetrans* inside the roots was changed by strain ExpR+ (Figure 3). The nematodes were significantly more aggregated in the roots of untreated or AttM treated plants compared to roots of ExpR+ primed plants (*P*-value < 0.0001). This suggested that primed plants responded more strongly to the early invasion of nematodes compared to non-primed plants, where nematodes easily established in the roots.

### Similar Effect of oxo-C14-HSL and BABA in Reducing *P. penetrans* Invasion Into Roots

**FIGURE 4:**
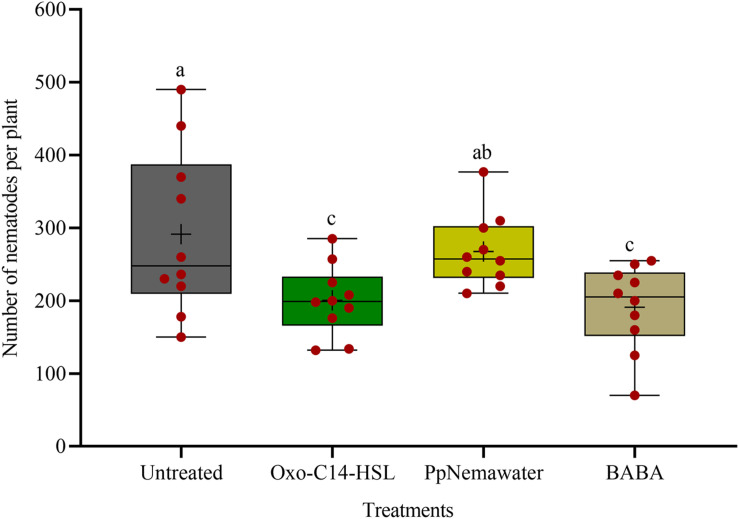
Effect of synthetic oxo-C14-HSL compared to DL-β-aminobutyric acid (BABA) and PpNemawater on the invasion of *Pratylenchus penetrans* into soybean roots. Different letters indicate significant differences among treatments (SAS proc GLIMMIX, Tukey adjustment, *n=10*).

We tested the efficacy of synthesic oxo-C14-HSL for defense priming, compared to the known priming inducer BABA and nematode associated molecules (PpNemawater). Stimulating soybean cv. Primus with oxo-C14-HSL or BABA led to a significant reduction of nematode invasion when compared to the control (GLM with Dunnet adjustment for multiple comparisons to the control, *P*-value < 0.005; Figure 4). Both compounds did not significantly differ in their reduction effect on nematode invasion into soybean roots. The nematode number in soybean roots stimulated with PpNemawater did not significantly differ from the control (*P*-value = 0.487). No significant effect on root fresh weight was observed within the experimental period, although a trend for a slightly better growth was observed for roots treated with oxo-C14-HSL (Supplementary Figure 2).

### Oxo-C14-HSL Systemically Induced Plant Defense Against *P. penetrans*

**FIGURE 5:**
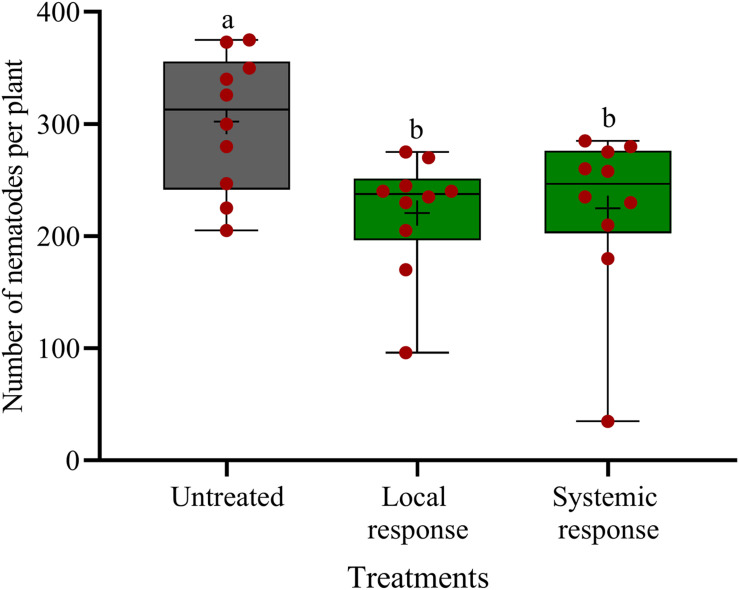
Systemic and local effects of the oxo-C14-HSL-producing *Ensifer meliloti* ExpR + strain on the invasion of *Pratylechus penetrans* into soybean roots. Different letters indicate significant differences among treatments in Tukey’s test (*n* = 10). Mean numbers of invaded nematodes in roots are shown as (+) for each treatment, while the medians are shown as (–).

In split-root systems, we investigated whether the observed effect of oxo-C14-HSL on nematode invasion is a local effect or mediated by a systemic plant response. When strain ExpR+ is inoculated on the same side of the split-root system as the nematodes, then it may affect the nematodes by both, systemic induction of plant defense and local interactions. Locally, there may be a direct antagonism against the nematodes or local plant responses like production of ROS or cell wall thickening in the affected root tissue. When strain ExpR+ is inoculated on the opposite side of the split-root system as the nematodes, then only one of both mechanisms can reduce the invasion of the nematode on the other side. Thus, a similar effect in both situations indicated a systemic plant-mediated mechanism and a negligible local interaction. The oxo-C14-HSL-producing *E. meliloti* strain ExpR+ was inoculated to the rhizosphere in one-half of the split-root system. Infective stages of *P. penetrans* were inoculated either to the side of the roots colonized by strain ExpR+ or to the opposite side, and counted in roots after 7 days. *P. penetrans* invasion into the roots was equally reduced on both sides of the roots of primed plants compared to the control without strain ExpR+ (Figure 5). Compared to the bacteria-free control, the total number of nematodes was significantly lower on both sides, with strain ExpR+ (223 ± 53, *P*-value < 0.0001) and on the opposite side without ExpR+ (225 ± 75, *P*-value = 0.0092). The additional reduction in median numbers of nematodes in the root due to co-localization of ExpR+ and *P. penetrans* compared to localization on opposite roots was minor and not significant (*P*-value = 0.88). This suggested that *E. meliloti* ExpR + systemically induced plant defense against *P. penetrans*, and that direct antagonism of strain ExpR + against *P. penetrans* or an induction of local root defense by strain ExpR+ did not significantly contribute to the suppression of the nematode. Roots infected with nematodes did not significantly differ among treatments after the experimental period. A trend of higher root mass was observed for roots that were locally inoculated with strain ExpR+. Overall, drenching soybean roots with *E. meliloti* strain ExpR + slightly increased the plant growth (Supplementary Table 1).

When testing direct toxic effects of the bacterial strains or synthetic oxo-C14-HSL against *P. penetrans*, only 3.0% of the tested nematodes were affected. This did not significantly differ from the MgCl_2_ control or the acetone control, respectively (Supplementary Table 2).

### Enhanced Defense of Plants Against *P. penetrans* Persisted After Removal of Priming Agents

**FIGURE 6:**
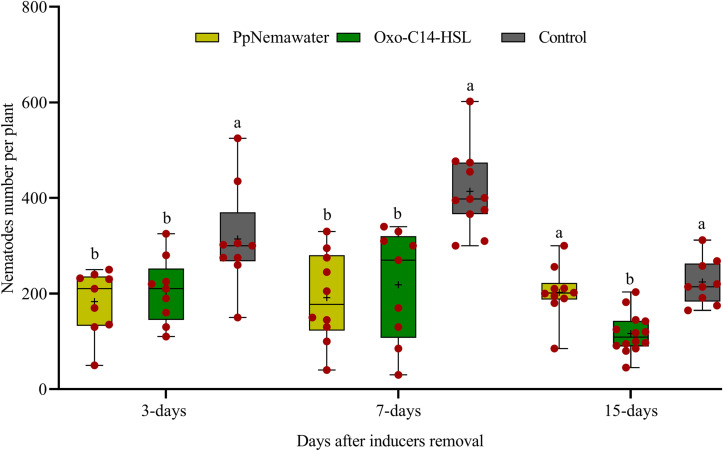
Persistence of enhanced defense of plants against root invasion of *Pratylenchus penetrans* after removal of inducing agents (oxo-C14-HSL or PpNemawater). Different letters indicate significant differences among treatments in Tukey’s test (*n* = 10).

We tested whether a temporary exposure of the root to oxo-C14-HSL was sufficient to result in reduced root invasion of the nematodes 3, 7, or 15 days after removal of the trigger. In parallel, we tested whether such a priming of plant defense was triggered by signaling compounds released from the surface of the nematode that simulated biotic stress. Soybean plants inoculated with oxo-C14-HSL were significantly less invaded by *P. penetrans* compared to control plants that were not exposed to oxo-C14-HSL (Figure 6). This priming effect stayed significant 3, 7, and 15 days after removal of oxo-C14-HSL. Similarly, the invasion of nematodes into soybean roots that have been exposed to surface-released compounds of *P. penetrans* was significantly lower compared to control plants, 3 and 7 days after removal of the inducer. After 15 days, the reduction compared to the control was statistically not significant.

### Priming Was Associated With Enhanced Synthesis of Phytoalexins and Phenolic Compounds in Roots Upon *P. penetrans* Challenge

**FIGURE 7:**
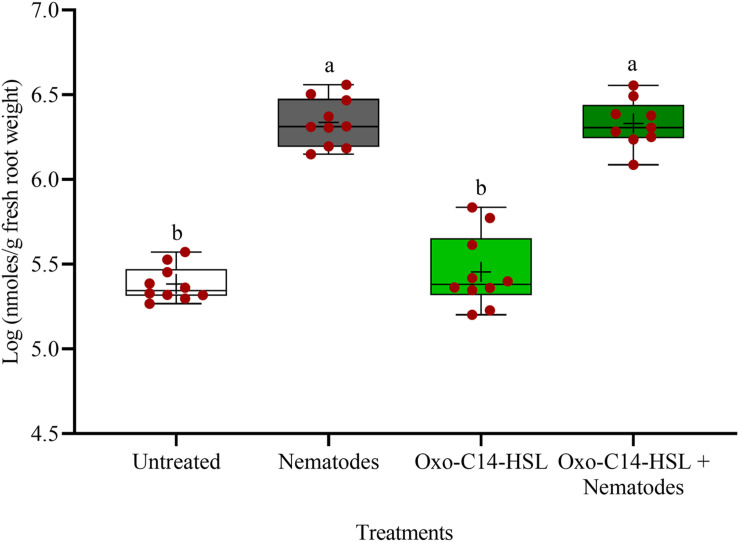
Quantification of coumestrol-like phytoalexins in roots of oxo-C14-HSL primed and non-primed plants of soybean cv. Primus by high-performance liquid chromatography. The mean of phytoalexin content in roots in soybean roots is shown as (+) for each treatment, while the medians are shown as (–). Different letters indicate significant differences among treatments in Tukey’s test (*n* = 10).

**FIGURE 8:**
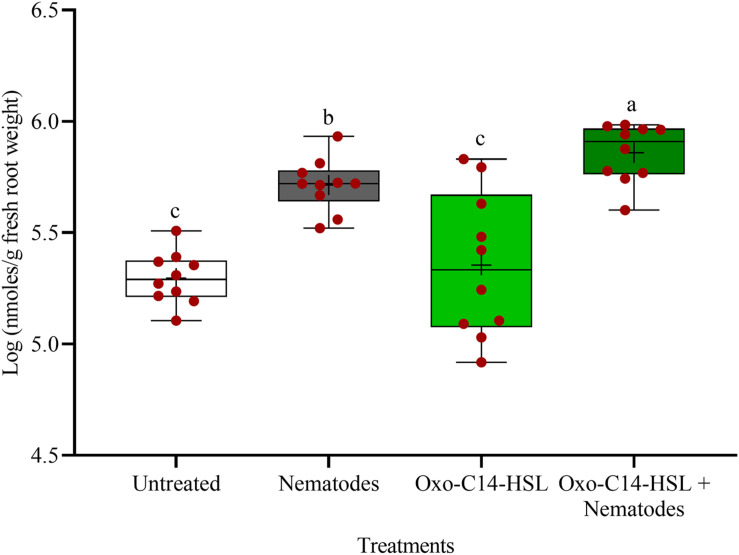
Quantification of glyceollin I content in roots of oxo-C14-HSL primed and non-primed plants of soybean cv. Primus by high-performance liquid chromatography. The mean of glyceollin content in roots is shown as (+) for each treatment, while the medians are shown as (–). Different letters indicate significant differences among treatments in Tukey’s test (*n* = 10).

**FIGURE 9:**
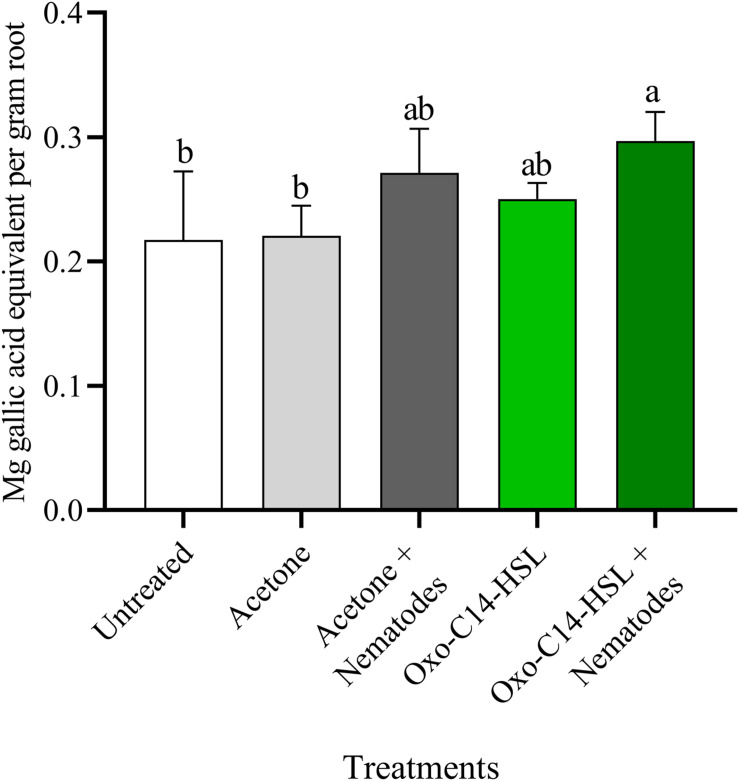
Quantification of total phenolic compounds in roots of oxo-C14-HSL primed and non-primed plants of soybean cv. Primus. Total phenolic compounds were quantified according to the Folin-Ciocalteu method by spectrophotometry at an absorbance of 765 nm and normalized to gallic acid. Different letters indicate significant differences among treatments in Tukey’s test (*n* = 5).

The phytoalexins in roots after exposure to oxo-C14-HSL and after invasion of *P. penetrans* into primed and non-primed roots were monitored by HPLC. Glyceollin I and coumestrol-like isoflavonoids were detected and quantified (Supplementary Figure 3). Without nematode invasion, the concentration of coumestrol-like compounds in primed and non-primed roots did not significantly differ, indicating that priming did not lead to an increased stress level to the plants (Figure 7). After nematode invasion, the phytoalexins coumestrol and glyceollin increased in concentration in the roots compared to control plants without nematodes. Most interestingly, upon nematode challenge, glyceollin synthesis was significantly enhanced in oxo-C14-HSL primed roots compared to non-primed roots (Figure 8). This suggested that the priming of soybean plants led to a more rapid and strong defense when inoculated with nematodes. In accordance, the oxo-C14-HSL primed roots showed a trend for a higher accumulation of total phenolic compounds compared to the acetone control after challenge with nematodes (Figure 9). However, measurement of total phenolic compounds was not sensitive enough to reveal significant differences between primed and non-primed roots before or after nematode invasion.

## Discussion

Rhizosphere inoculation of soybean cv. Primus with the *N*-acyl homoserine lactone oxo-C14-HSL producing strain ExpR+ reduced invasion of *P. penetrans* into roots. In contrast, rhizosphere inoculation of the nearly isogenic strain AttM, which expressed a lactonase to remove any synthesized oxo-C14-HSL, led to significantly more nematodes in the root. The pure compound oxo-C14-HSL also enhanced the plant defense; thus, other strain-related factors like exopolysaccharide production could not explain the observed effect. When the compound and nematodes were added to opposite parts of a split-root system, priming of plant defense was also achieved. This showed that the nematodes were not directly affected by oxo-C14-HSL or by local root responses to the compound. Thus, oxo-C14-HSL acted as a signaling molecule that systemically induced priming of the plant defense against *P. penetrans*. In a study by Zarkani et al. (2013), oxo-C14-HSL but not a short chain *N*-acyl homoserine lactone systemically induced resistance against leaf infection by the bacterium *Pseudomonas syringe* in *Arabidopsis thaliana*. In other studies on airborne diseases, oxo-C14-HSL induced systemic resistance of barley against the powdery mildew fungus *Blumeria graminis* f. sp. *hordei* (Shrestha et al., 2019) or the leaf rust causing fungus *Puccinia hordei* (Wehner et al., 2019) in a barley-genotype-dependent manner. Induction of barley by oxo-C14-HSL resulted in activation of MAP kinases, up-regulation of defense-related genes, and accumulation of lignin. Such basic defense reactions and strengthening of cell walls are likely to affect root penetration of *P. penetrans* as well.

Earlier studies used leaves from plants, which were exposed to oxo-C14-HSL, to test for a primed state of the plant in defense against the respective pathogen or pest (Zarkani et al., 2013; Shrestha et al., 2019; Wehner et al., 2019, 2021), without a time lag between oxo-C14-HSL exposure and biotic challenge. Our study showed that enhanced defense of the plants against root invasion of *P. penetrans* persisted after removal of the inducing compound for at least 2 weeks. This priming of the plant’s defense system was equally achieved by compounds released by the nematodes and the bacterial signaling compound oxo-C14-HSL. While perception of the nematode by the root and the following preparation of the plant for a nematode attack make sense ecologically, the priming by the bacterial inducer is less perspicuous and implies a cooperation among plants and rhizobacteria. Mostly, studies to chemically induce plant resistance or priming used compounds that mimic plant metabolites like β-aminobutyric acid, salicylic acid, pipecolic acid, or jasmonic acid (Navarova et al., 2012; Zhong et al., 2014; Ji et al., 2015; Hu et al., 2017; Thevenet et al., 2017). Depending on the concentration, such compounds may significantly stress the plant by activation of basal defense mechanisms like ROS accumulation, lignin formation, and callose deposition (Ji et al., 2015). In contrast, priming involves a memory of the plant and avoids ongoing stress and waste of resources. Priming of soybean plants was associated with enhanced synthesis of the isoflavonoid phytoalexin glyceollin upon *P. penetrans* challenge. Rapid production of glyceollin in soybean roots was shown to protect the roots from attack of the fungus *Fusarium solani* f. sp. *glycines* that causes sudden death syndrome (Lozovaya et al., 2004). The importance of rapid glyceollin synthesis was also shown for the defense of soybean plants against the pathogens *Macrophomina phaseolina* and *Phytophthora sojae* by comparing the susceptibility of transgenic plants with suppressed glyceollin synthesis to the wildtype (Lygin et al., 2013). Upon penetration of soybean roots by the soybean cyst nematode *Heterodera glycines*, glyceollin accumulated locally in roots of resistant but not in susceptible soybean cultivars (Huang and Barker, 1991). These plants were not primed and accumulation of glyceollin in roots of the resistant cultivar took 24 h. In our study, upregulation of the defense marker glyceollin by oxo-C14-HSL was not observed directly after the priming phase, but reached higher concentrations in primed plants than in non-primed plants after root invasion of the nematode. This observation corresponded to the model of defense priming in the narrow sense, where the priming stimulus is not causing a strong defense response of the plant but activates a memory mechanism that leads to a faster and stronger defense response upon subsequent challenge by a pathogen (Mauch-Mani et al., 2017). The primed state of the plant allows for a fast defense response, which is crucial for a successful defense against plant-parasitic nematodes. During root invasion, these nematodes start to suppress immune responses of the host locally. However, when the plant is faster in activating defense responses than the nematode can suppress innate immunity, then it may boost basal defenses using systemic signaling pathways and subsequently stop the attack (Topalović et al., 2020). The mechanism of defense priming in this study remains to be investigated. In isogenic soybean lines differing in susceptibility to soybean cyst nematodes, significant differences in DNA methylation between resistant and susceptible lines were observed that regulated miRNA functions during nematode–soybean interaction (Rambani et al., 2020). Studies showing dynamic epigenetic changes that play a role in plant-nematode interaction have recently been reviewed (Hewezi, 2020), but evidence for epigenetic changes of the plant to prime its innate immunity for enhanced resistance to plant-parasitic nematodes is lacking. The molecular mechanisms on how the plant memorized the priming event and activated defense still need to be investigated.

Interestingly, the dispersion pattern of invaded nematodes varied between primed and non-primed plants. Dense clusters of nematodes were observed in the root tissue of non-primed plants, in contrast to a significantly more even distribution of the nematodes in ExpR+ primed roots. Root-lesion nematodes explore the local physiological state of the root tissue by thrusting the stylet several times into a cell before entering the root (Kurppa and Vrain, 1985; Karakas, 2009). In non-primed roots, many nematodes may enter at a favorable root area or even the same entry site, thus leading to aggregated clusters of parasitic nematodes in the root. In primed plants, the fast local induction of plant defense may distract followers from entering at the same side, which resulted in a more even dispersion of the nematodes in the root.

Defense priming of barley cultivars against two airborne fungal pathogens was proposed to be genotype-specific and thus amenable to exploitation in breeding programs (Shrestha et al., 2019; Wehner et al., 2019). Differences in primability of soybean cultivars for induced defense against the soilborne root lesion nematodes still need to be investigated.

In conclusion, we showed that chemical signals in the rhizosphere can be harnessed to prime immunity of the soybean cultivar Primus against the root lesion nematode *P. penetrans*. The defense priming was systemically induced in the plant, did not lead to accumulation of defense markers prior to nematode attack, and persisted after removal of the priming agent. These results may have implications for future sustainable crop production, in which microbes could replace or complement chemical plant protection. Strengthening the primability of soybean varieties by marker-assisted breeding could become an environmentally friendly strategy of plant protection. As a vision, future cultivars may sensitively perceive signals from associated rhizobacteria that help to anticipate nematode attack, and rhizodeposits of the cultivar together with agricultural soil biome management may help to strengthen the bacterial priming inducers within the rhizosphere community of such a cultivar.

## Data Availability Statement

The original contributions presented in the study are included in the article/Supplementary Material, further inquiries can be directed to the corresponding author/s.

## Author Contributions

HH, AE, and LB designed the research plan. VH contributed with his expertise on soybean, chose and provided plant material, and discussed the experimental design and results. SA and AE performed the experiments. SA and BL analyzed phytoalexins. HH and AE did the statistical analyses. AE prepared the figures. SA wrote the manuscript. All authors revised the manuscript, contributed to the article, and approved the submitted version.

## Conflict of Interest

The authors declare that the research was conducted in the absence of any commercial or financial relationships that could be construed as a potential conflict of interest.
