# Supplementary material for: Priming Soybean cv. Primus Leads to Successful Systemic Defense Against the Root-Lesion Nematode, Pratylenchus penetrans
Source: Front Plant Sci. 2021 May 12;12:651943. doi: 10.3389/fpls.2021.651943 (PMC8149786; doi:10.3389/fpls.2021.651943)

Supplementary Material

Priming soybean cv. Primus leads to successful systemic defence against the root lesion nematode, *Pratylenchus penetrans*

Shimaa Adss[1], Benye Liu[2], Ludger Beerhues[2], Volker Hahn[3], Holger Heuer[1], Ahmed Elhady[1,4]
[1] Institute for Epidemiology and Pathogen Diagnostics, Julius Kühn-Institute (JKI) - Federal Research Centre for Cultivated Plants, Braunschweig, Germany
[2] Institute of Pharmaceutical Biology, Technische Universität Braunschweig, Braunschweig, Germany
[3] State Plant Breeding Institute, Universität Hohenheim, Stuttgart, Germany
[4] Department of Plant Protection, Faculty of Agriculture, Benha University, Benha, Egypt

Correspondence:
Dr. Ahmed Elhady, JKI, EP, Messeweg 11-12, 38104 Braunschweig, Germany
ahmed.gomaa@julius-kuehn.de

Supplementary Table 1: Local and systemic effect of the rhizobacterium *Ensifer meliloti*, producing *N*-3-oxo-tetradecanoyl-L-homoserine lactone, on growth of soybean plants (n = 10)

Treatments	Shoot weight (g)	Root weight (g)		Total root weight (g)	Total plant weight (g)
		Inducer side	Responder side		
Control	2.00 ± 0.48	0.63 ± 0.35	0.63 ± 0.32	1.25 ± 0.67	3.25 ± 1.16
Local response	2.44 ± 0.36	0.68 ± 0.19	0.84 ± 0.23	1.52 ± 0.43	3.96 ± 0.80
Systemic response	2.17 ± 0.33	0.65 ± 0.33	0.63 ± 0.23	1.28 ± 0.57	3.45 ± 0.90

Supplementary Table 2: Effect of *Ensifer meliloti* ExpR+, *Ensifer meliloti* AttM, or 6 µM *N*-3-oxo-tetradecanoyl-L-homoserine lactone (oxo-C14-HSL) on mortality of *Pratylenchus penetrans*, shown as percentage of dead nematodes (n = 24).

Bacterial strains effect			Oxo-C14-HSL effect	
Ensifer meliloti ExpR+	*Ensifer meliloti* AttM	$MgCl_2$ control	Oxo-C14-HSL	Acetone control
3.06 ± 0.37	3.00 ± 0.38	2.81 ±0.38	2.84 ±0.34	2.88 ±0.35

Supplementary Fig.1. Effect of priming by *N*-3-oxo-tetradecanoyl-L-homoserine lactone (oxo-C14-HSL) on the fresh weight of soybean roots.

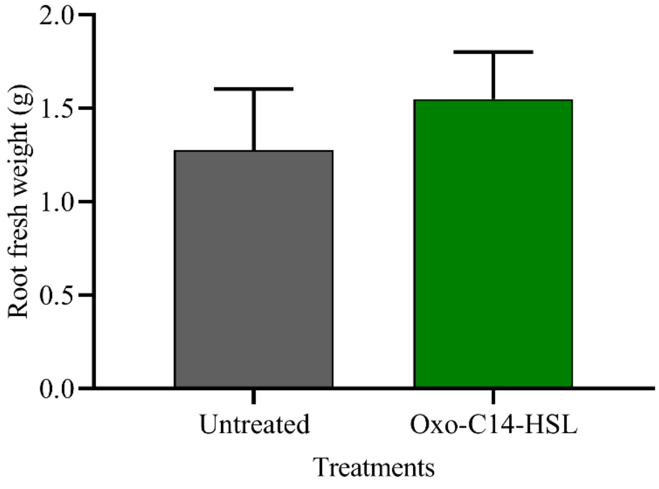

Supplementary Fig. 2. Effect of priming by *N*-3-oxo-tetradecanoyl-L-homoserine lactone (oxo-C14-HSL) compared to DL-β-aminobutyric acid (BABA) and compounds released from the surface of *Pratylenchus penetrans* (PpNemawater) on the fresh weight of soybean roots.

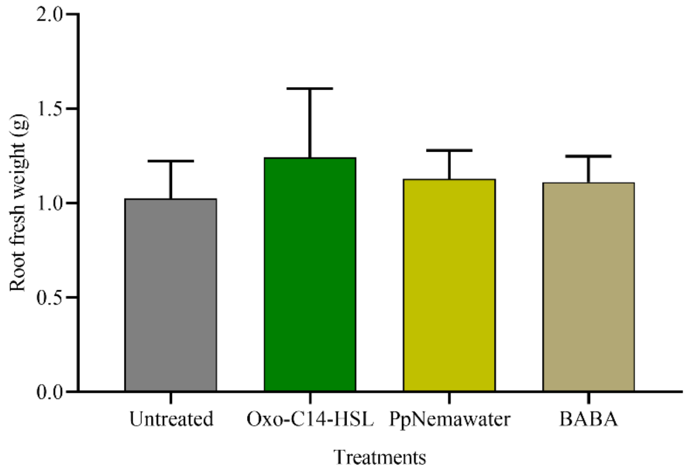

Supplementary Fig. 3. HPLC analysis of phytoalexins induced in roots of soybean cv. Primus that were either primed by *Ensifer meliloti* ExpR+ or non-primed ($MgCl_2$) upon root invasion of the nematode *Pratylenchus penetrans*.

Glyceollin, Ab =285nm

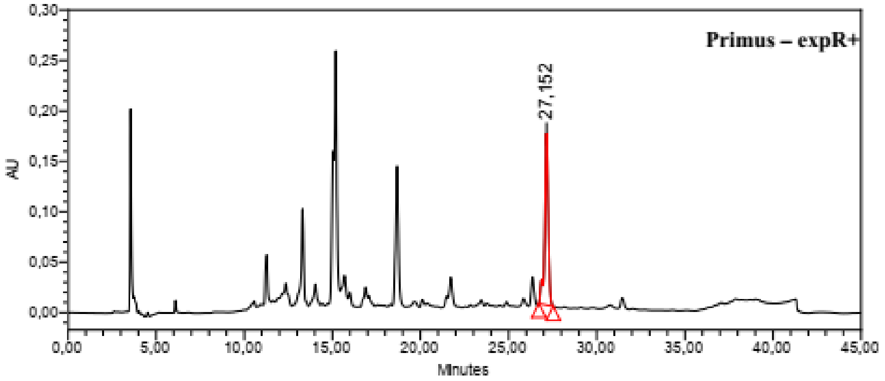

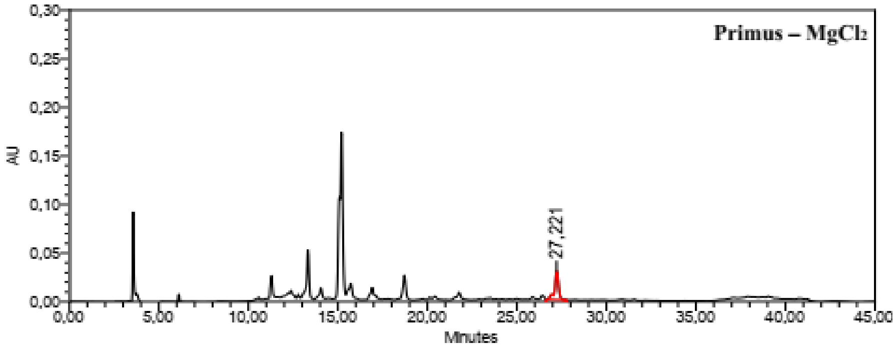

Coumestrol Ab= 343nm

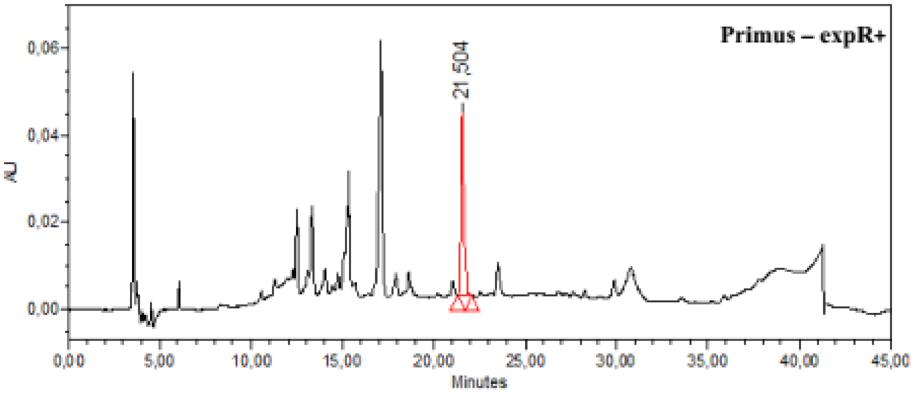

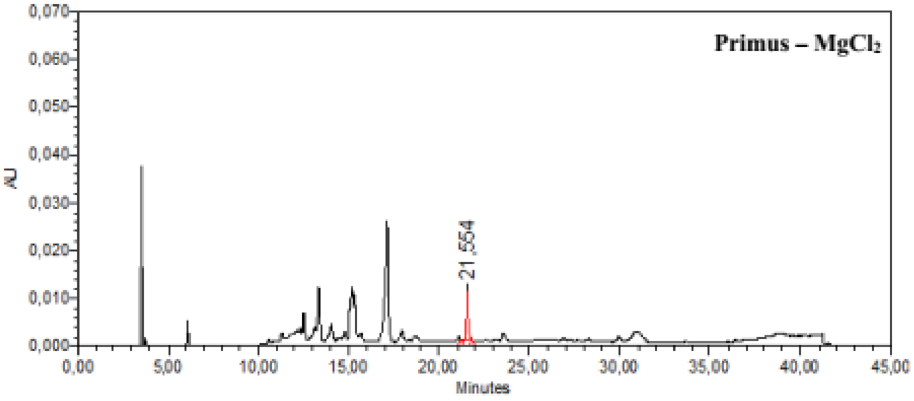

Supplement: Supplementary file 1 [file Data_Sheet_1.pdf]
